# Oropharyngeal Dysphagia as the Main Expression of Amyotrophic Lateral Sclerosis

**DOI:** 10.3390/medicina58050647

**Published:** 2022-05-09

**Authors:** Margarita Rugaitienė, Gytė Damulevičienė, Vita Lesauskaitė, Ingrida Ulozienė

**Affiliations:** 1 Clinical Department of Geriatrics, Lithuanian University of Health Sciences, 44307 Kaunas, Lithuania; gyte.damuleviciene@lsmu.lt (G.D.); vita.lesauskaite@lsmuni.lt (V.L.); 2 Department of Otorhinolaryngology, Lithuanian University of Health Sciences, 44307 Kaunas, Lithuania; ingrida.uloziene@lsmuni.lt

**Keywords:** oropharyngeal dysphagia, motor neuron disease, amyotrophic lateral sclerosis, fiberoptic endoscopic evaluation of swallowing, older patients

## Abstract

Amyotrophic lateral sclerosis (ALS) is the most common form of motor neuron disease. Only about 10% of ALS patients survive more than 10 years. Clinical studies show that multidisciplinary care statistically significantly improves survival compared to neurological care. ALS tends to manifest as limb weakness, but some patients present with bulbar symptoms, such as dysphagia and dysarthria. In rarer cases, the main symptom of ALS is oropharyngeal dysphagia. Respiratory muscle weakness is a relatively rare symptom at the onset of this disease and may lead to a fatal outcome due to aspiration pneumonia within about 1.4 years. These reasons led to a particularly complicated diagnosis of ALS in a 66-year-old Caucasian female patient complaining of dyspnoea and coughing while drinking water. Notably, dyspnoea is only present in one out of four treatment-seeking patients, and the course of ALS is non-specific. For these reasons, the diagnosis took an entire year while the patient underwent many tests and visited many specialists. However, the diagnosis was only made at a late stage of the disease. At present, the patient is almost unable to swallow food, water, or saliva, and is at a very high risk of aspiration, but refuses to have a percutaneous endoscopic gastrostomy performed. The objective of this case report is to highlight the fact that a symptom as simple as difficulty swallowing may be the result of severe disease, a frequent outcome of which is death.

## 1. Introduction

Motor neuron disease (MND) is a neurological disorder that damages motor neurons in the brain and the spinal cord. Amyotrophic lateral sclerosis (ALS) is the most common form of MND. The course of this disease leads to muscle weakness, paralysis, and death [1]. The disease occurs throughout adult life, with the peak incidence between 50 and 75 years of age, and is more common in men [2]. The precise aetiology of the disease is unknown. The development of this disease is determined by genetic and environmental factors (gender, geographic region, smoking) [3]. Currently, there are three popular theories regarding the factors behind the development of the disease—these are genetic factors, oxidative stress, and glutamatergic toxicity leading to dysregulated metabolism of important proteins. Specific treatment of ALS involves riluzole, which has been approved in the USA, Australia, and many European states. Riluzole is prescribed with the expectation of prolonging one year of the patient’s life by 2–3 months. ALS treatment is always symptomatic [4].

ALS tends to manifest as limb weakness, but some patients present with bulbar symptoms, such as dysphagia and dysarthria. As the disease progresses, over 50% of patients experience cognitive and behavioural disorders [5]. In rarer cases, the main symptom of ALS is oropharyngeal dysphagia (OD), which is hindered swallowing of food from the oropharynx to the proximal oesophagus. Common symptoms of OD include coughing, dyspnoea, choking, difficulty initiating a swallow, food being stuck in one’s throat, sialorrhea, nasal regurgitation, changes in voice or speech (e.g., hoarseness), weight loss, and changes in eating habits [6].

Studies have shown that dysphagia in patients with ALS correlates significantly with bulbar onset and with oral swallowing impairment [7]. In addition to causing OD malnutrition and dehydration, OD also increases social isolation, as patients experience constant fear and discomfort while eating and drinking, particularly in public places, and there is a decline in mental health, with OD patients being more prone to depression [8].

Medical evaluation involves radiological and laboratory examination and is based on the principle of ruling out other diseases [9]. One of two gold standard methods of diagnosing OD is the fiberoptic endoscopic evaluation of swallowing (FEES) [10]. This method is useful for detecting swallowing deficits and laryngeal sensitivity in patients with ALS [7]. FEES enables the specialist to study the physiology of swallowing; evaluate the type and severity of OD; and develop a plan for further evaluation, dietary recommendations, and rehabilitation [11].

The main objective in OD treatment is to create a modified diet plan adjusted for the individual patient. The diet has three main goals—reducing the risk of aspiration, ensuring proper nutrition, and ensuring proper eating according to the general status and swallowing function of the patient. If the individual’s swallowing safety and efficiency cannot reach an adequate level of function, or if swallowing function does not adequately support nutrition and hydration, the specialist may recommend alternative routes of intake (e.g., nasogastric tube, gastrostomy) [12]. Once dysphagia severity and aspiration risk are evaluated via FEES, a safe level of liquid viscosity and solid food consistency can be determined.

Furthermore, electrical stimulation and exercises of the swallowing muscles have been shown to be efficient. Exercises induce changes in the physiology of swallowing, either by improving sensory-motor integration or by gaining voluntary control over the timing or coordination of selected oropharyngeal movements during swallowing. Swallowing therapy may also involve neuromuscular exercises to increase tongue strength, endurance, power, and range of motion. Different exercises are intended to address different aspects of impaired swallowing [13].

## 2. Case Report

A 66-year-old woman began to experience disordered speech, exhausting spells of dyspnoea, choking on liquids, gradual weight loss (5 kg in 3 months), and sialorrhea. The patient sought neurological consultation two months after symptom onset. The neurologist conducted a neurological examination and decided to perform magnetic resonance imaging (MRI). Conducted in October 2020, the MRI scan showed a small lesion in the cerebellum. Having analysed the image, the neurologist referred the patient for a neurosurgical consultation. The neurosurgeon consulted the patient regarding a suspected oncological process in the brain. A team of neurosurgeons and radiologists decided that there were not enough findings to indicate an oncological disorder, while the lesion was gliotic, and therefore, surgical or other treatment was not necessary. However, as her symptoms worsened, the patient consulted an otolaryngologist 8 months later. A thorough examination revealed left vocal fold paresis, as the fold remained at an intermediate position with minimal movement observed. The otolaryngologist, believing the paresis to have been caused by an oncological process in the neck or mediastinum area, referred the patient for a pulmonological consultation. A few days later, the pulmonologist conducted pulmonary function tests (spirometry results were normal—FVC 2.49 (95%); FEV1 2.24 (102%); FEV1/FVC 0.902 (119%); Epworth scale—3 points) and performed auscultation. Objectively, severe dysarthria was observed. The pulmonologist and the otolaryngologist agreed to refer the patient for computed tomography (CT) of the neck and chest. The CT scan showed mild asymmetry in the palatine tonsils (the left one was larger), with uneven distribution of the contrast medium (c/m). The lingual tonsil was unevenly enlarged, with even c/m distribution and a narrow stripe of c/m accumulation in the anterior right vallecula. There were no abnormalities of the epiglottis. Aryepiglottic folds and the sinus pyriformis were distinct and symmetrical, with no structural changes. Salivary glands were symmetrical and of normal structure. Vocal folds were symmetrical and evenly contoured, and the glottis was not deformed. Laryngeal ventricles were symmetrical. There were no abnormalities in the subglottic space. Laryngeal cartilages were of normal structure. The thyroid was of normal size, with symmetrical lobes and even distribution of the c/m. Sinuses were clear. There were no lesions or signs of pathological c/m distribution in the visible mediastinum. Cervical lymph nodes of the IIA group were smaller than 0.8 × 1.0 cm. Nodes of the IIIB group were small. Therefore, the results did not reveal any pathological structures or unusual c/m distribution, and once again, there were not enough findings to signify any oncological process.

However, the patient’s symptoms continued to worsen. Her speech became incomprehensible, there was no more articulation, and she experienced constant sialorrhea and choking. The patient’s ability to eat continued to worsen, as did her social isolation and depressiveness. In May 2021, the patient was hospitalised at the Neurological Department of the Hospital Lithuanian University of Health Sciences (LSMU) Kaunas Clinics due to worsening neurological symptoms. The patient underwent new and repeated tests and procedures, including transcranial magnetic stimulation (TMS), electromyography (EMG), and magnetic resonance imaging (MRI) of the brain combined with tractography. During the medical team conference, the neurologists concluded that the patient’s symptoms and test results were compatible with the diagnosis of ALS. The patient was prescribed riluzole 50 mg twice daily and referred for rehabilitation therapy.

**Figure 1:**
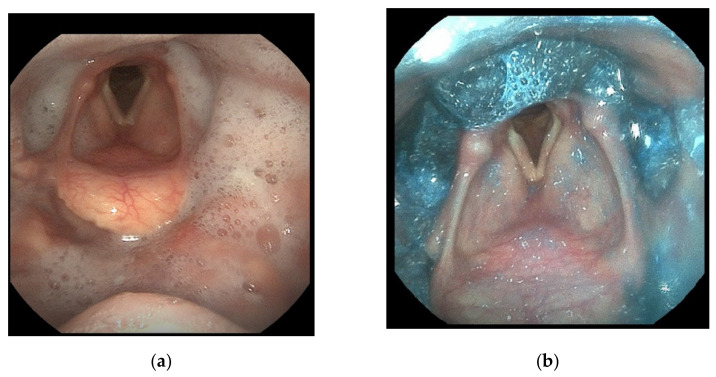
Thin liquid (milk) (**a**) and moderately thick liquid (**b**) samples revealed delayed bolus transit to the pharynx. Residua in the vallecula, pyriform sinuses, and ventricles.

In July 2021, the patient was hospitalised at the Geriatric Department of LSMU Kaunas Hospital for further evaluation and treatment of OD. At that time, her speech was completely incomprehensible, and she had difficulty swallowing even solid food. Upon assessing the woman’s vital signs and laboratory blood tests, no significant pathological changes were observed, ALSFRS-R–39 points. The patient’s quality of life was assessed using the Dysphagia Handicap Index (DHI) [14], Swallowing Quality of Life (SWAL-QoL/SWAL-CARE) [15], and EAT-10 [16] questionnaires. The patient scored 54 on the DHI questionnaire, which indicates dysphagia having a severe impact on quality of life. The SWAL-QoL/SWAL-CARE and EAT-10 questionnaires showed that the patient’s quality of life was poor due to the disease. FEES revealed laryngeal asymmetry at rest, hypersalivation, partial tongue base retraction, and a moderate response in the laryngeal sensation touch test. Milk and food samples revealed delayed bolus transit to the pharynx. The patient had to perform 5–10 motions of swallowing to properly clear the pharynx. During bolus formation, food and saliva leaked from the mouth. The patient had a score of 6 on the Penetration-Aspiration Scale [17] (material enters below the vocal folds and there is an adequate response in the form of coughing and throat clearing) (Figure 1). The patient was prescribed combination treatment including thickened liquids at level 3 (pudding consistency) and electrical stimulation of the swallowing muscles at 7–9 mA for 40 min. To prevent malnutrition, percutaneous endoscopic gastrostomy (PEG) was suggested to ensure the patient’s nutrition, which the patient refused. Every day, the patient received individual therapy from a logotherapist and was taught exercises to strengthen the swallowing muscles. Treatment lasted for 10 days. After combination treatment, FEES was repeated and showed a clear improvement in the patient’s swallowing of a level 2 thickened liquid. The patient was contacted three weeks after treatment. In her subjective opinion, her swallowing had improved, and there had been fewer instances of choking. Considering that the disease is always progressing, the patient was offered PEG on a scheduled basis. Presently, the patient refuses to discuss having PEG performed. For now, the longevity of the improvement achieved by the treatment remains unclear.

## 3. Discussion

This clinical case is interesting in that the onset of the disease was marked by relatively rare symptoms. Typical symptoms of ALS include asymmetric limb weakness in 60–80% of patients, whereas bulbar muscle weakness occurs in only 1–9%, with jaw weakness, muscle atrophy, fasciculations, and cramping occurring in 5–7% of patients (8). In Korea, 500 cases of ALS diagnosed in 2007–2019 were investigated. Of these, 335 (67.0%) were patients with limb-dominant onset, 144 (28.8%) were patients with bulbar-dominant onset, and 21 (4.2%) were patients with respiratory-dominant onset. ALS diagnosis took 12.7 ± 11.9 months to establish. Men were diagnosed with the disease sooner than women [2].

What is more, the patient in this case report was 66 years old, putting her at a relatively lower risk of bulbar symptoms, while the overall risk of ALS at this age remains high. A retrospective cohort study of 916 ALS cases in Israel compared three age groups, in which 93 patients were ≤43 years old, 715 patients were 44–74 years old, and 108 patients were ≥75 years old. This study showed that bulbar symptoms are significantly more frequent in ≥75-year-old patients, most of which are women [18]. In the case reported here, the diagnosis took a relatively long time, during which the disease had progressed to a severe stage. Remarkably, however, liquid thickeners had been prescribed before the diagnosis was made. The patient was aware of the severity of her situation and complied with the doctor’s orders of using liquid thickeners and following a diet of soft mashed food to prevent aspiration pneumonia.

Approximately 45% of OD patients refuse to use liquid thickeners, and the outcome in 15% of those patients is death [19]. Importantly, patients with ALS and OD must change their eating habits and have the amount and consistency of their food adjusted. If there are marked changes in eating habits, patients may lose weight and develop malnutrition. Using the BMI to assess the nutritional status of these patients is rather complicated and not recommended. If only the BMI is used, the fat-to-muscle ratio is not taken into account. Therefore, even obese patients may have mild or severe malnutrition. In addition, OD patients tend to consume relatively more carbohydrates due to low chewing efficiency and/or disordered oral or pharyngeal swallowing phases. The syndrome of malnutrition is just as noteworthy as dehydration. Therefore, liquid thickeners and swallowing muscle exercises should be used as early as possible. Swallowing muscle weakness has a strong influence on the survival rate of ALS patients. This is firstly due to death caused by complications of aspiration pneumonia, and secondly due to death caused by starvation. According to research, tongue muscle weakness can be considered a prognostic factor for survival in ALS. What is more, an effective cough reflex is important in lowering aspiration risk [20]. The effectiveness of the cough reflex in the case reported here was moderate. The patient was advised to cough after every swallow of the bolus and to remain in a vertical position, rather than lie down, after eating.

In the case reported here, the disease manifested as bulbar symptoms. Patients with bulbar-type ALS tend to present with longer and more severe spells of coughing and choking in comparison to spinal-type ALS patients. The aforementioned symptoms may be provoked by anxiety. On the other hand, anxiolytic medications, which may be prescribed to prevent these spells, have also been found to provoke these symptoms [21].

After confirming the diagnosis of ALS, the patient was immediately prescribed riluzole. There is a recommendation to begin pharmacological treatment as early as possible, as there is a significant difference in the mortality rate between patients treated with riluzole and untreated patients [19]. The diagnosis of ALS tends to take a relatively long time, as ALS is not the first diagnosis suspected in cases worsening this rapidly. Diagnosing ALS requires a high level of knowledge and experience and is usually performed by ruling out other diseases.

It is important to note that electrical swallowing muscle stimulation for the patient is effective in treating patients with ALS. A study in Germany using pharyngeal electrical stimulation shows a statistically significant improvement in quality of life. Improvements in patients’ psychological status, appetite, and eating pleasure were observed in the analysis of SWAL-QoL questionnaires [22].

Although the diagnosis of ALS takes the same amount of time in people older than 80 years and those younger than 80 years, age does have an influence on patient survival—survival of <80-year-olds is significantly higher than in >80-year-olds. However, the patient’s sex and main symptoms (bulbar or spinal type) are not related to survival [23].

The most precise diagnosis of dysphagia, aspiration, and their dynamics is provided by videofluoroscopy and FEES. Such patients must undergo highly sensitive diagnostic testing that aims to detect even the slightest manifestation of the disease. If safe nutrition cannot be ensured, PEG must be performed [7]. Thus far, the patient in this case report has refused this procedure, but her decision may only be a question of time. Studies show that bulbar dysphagia patients undergo PEG more frequently than those with spinal onset [19]. In clinical practice, diagnostic questionnaires for dysphagia, the water-swallowing test, and other tests are less sensitive to small-scale changes in OD patients. A timely diagnosis of severe dysphagia and high aspiration risk combined with PEG decreases the risk of aspiration, asphyxia, and other complications.

## 4. Conclusions

In summary, ALS is a constantly progressing disease. The differential diagnosis of this disease is complicated by the fact that OD symptoms (caused by stroke, dementia, Parkinson’s disease, or xerostomia) are relatively common in older people. After analysing this clinical case, it is evident that the cause of oropharyngeal dysphagia must be clarified as early as possible. Preventing severe complications of this disease is important, but so is the patient’s quality of life. Studies have shown that early diagnosis and treatment of OD improves the quality of life and the outcome in ALS patients [24]. This case report illustrates the manifestation of ALS as OD and the long period of time taken to make the diagnosis, which was complicated further by the COVID-19 pandemic. With the closure of most outpatient services in the country, the patient had difficulty accessing specialists. Diagnosis of ALS under normal living conditions can take up to 6–12 months, and these numbers may increase during a pandemic.

## Data Availability

All the data are available from the corresponding author upon reasonable request.
